# Serial assessment of myocardial T2 in Duchenne muscular dystrophy

**DOI:** 10.1186/1532-429X-13-S1-P282

**Published:** 2011-02-02

**Authors:** Janaka Wansapura, Kan Hor, Wojciech Mazur, Robert Fleck, Michael Taylor, D Woodrow Benson, William Gottliebson

**Affiliations:** 1 Cincinnati Children's Hospital, Cincinnati, OH, USA; 2 Christ Hospital, Cincinnati, OH, USA

## Background

Duchenne Muscular Dystrophy (DMD), a lethal X-linked skeletal and cardiac myopathy, affects 1/3500 males[1-2]. MRI studies have shown occult ventricular dysfunction and myocardial fibrosis in DMD patients. Previously we used the Full Width of Half Maximum *(FWHM)* of T2 distribution in LV to quantify the myocardial structural heterogeneity in DMD patients. In DMD subject groups, we showed that *FWHM* of the T2 histogram rose progressively with age and decreasing EF indicating that functional impairments could be associated with pre-existing abnormalities in tissue structure in young DMD patients. In this study we assessed the T2 distribution in DMD patients at two time points. We hypothesized that serial FWHM changes can be detected in individual DMD patients during a time when left ventricular ejection fraction (EF) changes are insignificant.

## Methods

MRI Data of eighteen DMD patients obtained at two time points were analyzed (mean=12 years, range =8-18 years) with a mean time of 2.3 years between the studies. Spin echo images of the left ventricle in the short axis plane were acquired using a black blood dual spin echo method. Imaging parameters were: Slice thickness = 5mm, in plane resolution = 1.4mm×1.4mm, echo train length = 5, Echo times: TE_1_ = 6 ms, TE_2_ = 34ms. A histogram of LV T2 distribution with bin size equal to 1 ms was constructed for each subject. The Full Width at Half Maximum (*FWHM*) was calculated after applying box car averaging. The *FWHM* was defined as the width of the histogram at half the maximum height.

## Results and conclusions

In the interval between studies the *FHWM* increased in all but two patients (89%). The average increase in FWHM was 6.8 ± 3.9 ms. The mean T2 increased in 5 patients while it declined in 13 patients. In a previous study we showed that *FWHM* did not change with age in normal subjects. Thus increase in T2 heterogeneity quantified by *FWHM* indicates progression of disease over a relatively short period.

